# Serology as a Tool to Assess Infectious Disease Landscapes and Guide Public Health Policy

**DOI:** 10.3390/pathogens11070732

**Published:** 2022-06-27

**Authors:** Andrea H. Haselbeck, Justin Im, Kristi Prifti, Florian Marks, Marianne Holm, Raphaël M. Zellweger

**Affiliations:** 1 International Vaccine Institute, Seoul 08826, Korea; andrea.haselbeck@ivi.int (A.H.H.); justin.im@ivi.int (J.I.); kristi.prifti@ivi.int (K.P.); fmarks@ivi.int (F.M.); marianne.holm@ivi.int (M.H.); 2 Department of Microbiology and Parasitology, University of Antananarivo, Antananarivo 566, Madagascar; 3 Cambridge Institute of Therapeutic Immunology and Infectious Disease, School of Clinical Medicine, University of Cambridge, Cambridge CB2 0AW, UK; 4 Heidelberg Institute of Global Health, University of Heidelberg, 69120 Heidelberg, Germany

**Keywords:** infectious disease surveillance, serology, antibodies, disease etiology, LMIC

## Abstract

Understanding the local burden and epidemiology of infectious diseases is crucial to guide public health policy and prioritize interventions. Typically, infectious disease surveillance relies on capturing clinical cases within a healthcare system, classifying cases by etiology and enumerating cases over a period of time. Disease burden is often then extrapolated to the general population. Serology (i.e., examining serum for the presence of pathogen-specific antibodies) has long been used to inform about individuals past exposure and immunity to specific pathogens. However, it has been underutilized as a tool to evaluate the infectious disease burden landscape at the population level and guide public health decisions. In this review, we outline how serology provides a powerful tool to complement case-based surveillance for determining disease burden and epidemiology of infectious diseases, highlighting its benefits and limitations. We describe the current serology-based technologies and illustrate their use with examples from both the pre- and post- COVID-19-pandemic context. In particular, we review the challenges to and opportunities in implementing serological surveillance in low- and middle-income countries (LMICs), which bear the brunt of the global infectious disease burden. Finally, we discuss the relevance of serology data for public health decision-making and describe scenarios in which this data could be used, either independently or in conjunction with case-based surveillance. We conclude that public health systems would greatly benefit from the inclusion of serology to supplement and strengthen existing case-based infectious disease surveillance strategies.

## 1. Introduction and Scope of the Review

Public health surveillance can be defined as “the continuous and systematic collection, analysis and interpretation of health-related data needed for the planning, implementation and evaluation of public health practice” [1].

Infectious disease surveillance is an important public health tool that enables identification of epidemiological trends, risk groups, early detection of outbreaks and novel pathogens and impact assessment of interventions in defined populations [2]. Traditionally, surveillance of infectious diseases has been primarily “case-based”, i.e., tracking of infections through disease cases. As such, effective infectious disease surveillance requires close and continuous monitoring of health events, systematic and accurate confirmation of infectious disease etiology, as well as an effective system for data storage, management and use.

Today, even wealthy countries with integrated health systems, up-to-date census information and adequate access to healthcare are not always able to completely capture the burden and underlying epidemiology of diseases. This is even more challenging in low- and middle-income countries (LMICs), where public health systems are often inadequately equipped to conduct broad-scope investigations into disease etiology. Historically, externally-funded research programs assessing the burden of priority pathogens have implemented surveillance infrastructure in these settings, but surveillance efforts are often not maintained beyond the period of study [3].

Serology, the examination of serum for the presence of pathogen-specific antibodies, has long been used to assess individuals’ previous exposure and immunity to specific pathogens. However, it has been less utilized as a tool to evaluate infectious disease burden at the population level.

This article provides a case for the use of serology in infectious disease surveillance, with an emphasis on low- and middle-income countries (LMICs). First, we describe case-based surveillance and point to the challenges of implementing this type of surveillance in low resource settings. We then describe how serology is gaining momentum to understand the epidemiology of infectious diseases by making use of novel technologies, and we provide examples of where serology has been used to define disease burden. Finally, we synthesize how information gained from serology, either alone or in combination with case-based surveillance, can inform decision makers and guide public health policies.

## 2. Case-Based Infectious Disease Surveillance—Current Use and Limitations

### 2.1. Passive and Active Surveillance

Infectious disease surveillance can be divided into two main categories: passive surveillance and active surveillance [4]. Passive surveillance is an approach that detects infections when individuals with overt disease or symptoms seek medical attention at a health-care facility. Passive surveillance systems can include all facilities in a country or be limited to a selected network of sentinel sites providing healthcare to population subset [2,5]. In active surveillance infections are detected through proactive, systematic disease screening within a population [2,5].

Passive surveillance typically detects symptomatic cases, as only people with symptoms seek health care, while active surveillance can either enumerate overt cases or rely on other screening measures that may capture asymptomatic cases (e.g., systematic laboratory testing) [6]. Passive surveillance enables the assessment of the burden of an infectious disease to the health care system, while active surveillance measures the occurrence of infections within a population or community [7]. The latter includes asymptomatic and subclinical cases when detection does not rely on symptoms alone.

### 2.2. Implementing Surveillance—Challenges

Both active and passive surveillance approaches share several features.

#### 2.2.1. Definition of Catchment Population

The surveillance catchment population should be geographically well-defined; ideally population demographic information should be captured by a census or health and demographic surveillance systems (HDSS) and updated periodically [8]. The population under surveillance is the denominator for determining the rate of occurrence of defined health outcomes. Particularly in urban centers of poor countries, there is a large proportion of informal settlements and slum areas which experience high rates of migration [9]. Associated uncertainty about the size, distribution and demographics of a geographical region can thereby limit the extent to which credible inference about epidemiological trends can be made.

#### 2.2.2. Health Care Seeking Behavior

Active surveillance generates health data without requiring individuals to actively seek health care, because healthcare workers are dispatched to a community to gather health information from the general population [8,10]. This type of surveillance requires a significant amount of human and financial resources, and is not common implemented outside of well-funded research projects.

Passive sentinel surveillance is less resource intensive but relies on and is influenced by healthcare seeking behavior [10]. The decision to seek healthcare is influenced by many individual and external factors, such as disease severity, socio-economic status, seasonality and climate change, accessibility, availability of alternatives, education level and prior experiences [11]. Different contextual factors and behaviors between population subgroups may complicate the comparison of surveillance data between regions. Particularly in low-income settings with relatively high barriers to health care and/or lack of universal healthcare coverage, understanding and recording actual behavioral patterns is crucial to interpreting surveillance estimates [3]. As an illustration, health care surveys conducted in six sub-Saharan African countries as part of a multi-country standardized typhoid fever surveillance program revealed highly heterogeneous and overall limited health-care seeking behavior [12].

#### 2.2.3. Case Definitions

Symptoms of many tropical diseases are unspecific and often overlapping. In addition, in LMICs physicians often treat empirically due to financial, diagnostic and laboratory equipment or manpower limitations and/or because ascertaining etiology is not a priority. However, specific and standardized case definitions are essential for disease surveillance. Cases are generally categorized as *confirmed* cases (usually ascertained through laboratory procedures), *probable* cases (clinical features are indicative of the infection) and *suspected* cases (fewer or atypical clinical features) [13]. Of note, case definition often change over time and vary between regions, resulting in major challenges in the direct comparison of disease burden.

#### 2.2.4. Biological Specimen Collection and Diagnostics

Case confirmation typically implies the identification of the causative agent within the body via laboratory analysis of a biological specimen. Techniques include classical pathogen isolation and identification, detection and quantification of pathogen-specific antibody and/or antigens using serological tests, or molecular approaches for detection nucleic acid or genomic elements of a pathogen of interest. However, there are many challenges to collecting and handling biological specimens [14,15,16]. Obtaining samples from blood, bone marrow, cerebrospinal fluid or tissue is an invasive process and requires specialized equipment and trained staff. Collection of other samples, such as sputum, urine or stool, is less invasive, but there are programmatic challenges to sample collection and handling that can impact their utility. Further, correct pathogen identification in these non-sterile specimens can be challenging.

Ideally, cases should be ascertained using a “gold standard” diagnostic, but the gold standard may not always be readily available [17], especially in lower resource settings where the laboratory capacity is limited. Instead, many places rely on point-of-care rapid diagnostic tests which are easier to implement as they require limited equipment and training while enabling early treatment initiation and improving health outcomes [3,18].

## 3. Serology to Assess Disease Burden

### 3.1. Rationale for Using Serology to Assess Disease Burden

Serology is the science of blood serum and other body fluids. In the context of medicine, the term is traditionally used to describe the detection of antibodies in the blood serum but it can also be used to describe antibody measurements in other clinical samples of body fluids (e.g., saliva). Antibodies (also called immunoglobulins) are typically produced in response to exposure to pathogens or after vaccination and can recognize unique molecular targets from that pathogen (or vaccine). Antibodies are therefore often described as “specific” for a certain pathogen (e.g., influenza-specific antibodies, measles-specific antibodies). As part of the immune response, antibodies fulfill a range of protective functions which will not be discussed here. However, of particular relevance for the current discussion, antibodies can persist from months to years after an infection (or vaccination), and can therefore be seen as biomarkers of past exposure to various pathogens such as viruses, bacteria or parasites. Therefore, measuring antibodies informs about an individual’s (at least recent) history of exposure to pathogens, depending on the duration of the immune response.

The idea of using serology-based methods to measure the presence of pathogen-specific antibodies in populations of interest and define the level of exposure and infectious disease landscape is rapidly gaining traction [19,20,21,22,23,24]. It is increasingly seen as a powerful tool that could complement classical case-based disease surveillance, generate a wealth of information and help guide public health policy. While its use is already wide-spread in wealthier countries (e.g., Europe [25], Australia, US [21]), it is still under-utilized in LMICs. Below, we present some of the benefits and challenges linked to using serology for disease burden assessment.

### 3.2. Advantages of Using Serology-Based Techniques to Define Disease Burden

#### 3.2.1. Detection of Past Cases Regardless of Symptoms Occurrence

In contrast to case-based surveillance which consists of tallying symptomatic cases presenting to the healthcare system, serology measures historical pathogen exposure, and is independent of the current disease status. Therefore, sub-clinical cases and symptomatic individuals who do not seek health care are missed by case-based surveillance, which paints an incomplete picture of the disease burden. In contrast, serology-based methods can capture both symptomatic and asymptomatic cases, irrespective of their health seeking behavior, because their antibody profile will keep a trace of past exposure [20]. While case-based surveillance detects current conditions (and laboratory confirmation can only be performed while the etiologic agent is present), serology-based methods detect exposure “after the fact”, even once the pathogen has been cleared.

While antibody induction kinetics varies according to the pathogen, the first type of antibody produced after infection is usually the shorter-lived IgM, followed by the longer lasting IgG. At mucosal sites, the predominant antibody type is IgA. Antibody levels wane over time (and are boosted upon re-exposure), with IgM decreasing faster than IgG levels. Therefore, while serology typically gives no firm indication when in the past the infection took place, measuring the ratio and individual levels of IgM and IgG can give some clues about how recent the infection was. This has been performed, for example, for flaviviruses [26], hepatitis E [27] or SARS-CoV-2 [22].

#### 3.2.2. A Variety of Samples Can Be Used

A variety of samples can be used for antibody detection [24]. Serological antibody detection is primarily performed on serum, but antibodies can also be measured in eluted dried-blood spots (DBS) [28], in saliva or nasal swabs for mucosal or respiratory pathogens [29,30] and in cerebrospinal (CSF) fluid for neurotropic pathogens [31]. Saliva sampling is increasingly seen as an attractive option since it is easy and non-invasive [32,33,34,35], but quantifying antibody is complicated by the absence of standardized sampling methods [36] and inherent heterogeneity of saliva protein content across individuals [33]. Typically, very small sample volumes are necessary for antibody detection, in the order of the microliter [20]. Importantly, serology can be performed on frozen samples, opening the possibility of dissociating sample collection from sample analysis, and using convenience or archived sample collections.

#### 3.2.3. Use of Convenience Sampling and Historical Collections

Performing bespoke sero-surveillance studies, where a population is screened for the presence of antibodies of interest, is often time-, labor- and cost-intensive [19]. Thankfully, antibodies can be detected in serum, DBS and saliva using only a small volume of fresh or frozen sample, and therefore serology can be performed on convenience samples, such as residual serum from hospital laboratories [37], blood banks [38,39] and historical collections [23,40]. In these cases, proper sample preservation is crucial to guaranteeing scientific validity of the results, and a rigorous ethical framework must be in place to ensure that consent for further testing of the samples has been received [20]. As illustrated in Vietnam, a hybrid sample collection approach combining the expansion of existing studies with convenience sampling can also be adopted to balance biases inherent to each collection technique [19].

#### 3.2.4. Multiplexing

Surveillance is often focused on a single pathogen of interest, in particular for project-based activities, which are usually designed to address a specific research question. Serology is well suited for multiplexing, allowing for the simultaneous detection of tens of pathogens at the same time, using the same sample. Future development might even expand this number to thousands (see Section 4. Current Methods, New Technologies and Future Directions) [41]. Multiplexing is compelling not only because it decreases the “per pathogen” cost of analysis, but it also generates a much more comprehensive picture of the infectious disease landscape. Coupled with geo-localization (when possible), longitudinal sampling and modern data computational techniques, this approach could generate a wealth of information about the spatio-temporal distribution of antibodies and infectious diseases to inform public health decision making [20,23].

Antigen-multiplexing is also possible (either across pathogens or using different epitopes of one pathogen) and has long been used for hepatitis B to differentiate between immunity due to natural infection vs. vaccination or even distinguish various phases of infection (acute vs. chronic) [42]. As vaccine coverage expands and novel vaccines are being rolled out, there is a growing need for serology tests that can reliably differentiate between natural infection and vaccination in order to disentangle antibodies induced by vaccination (a measure of vaccine coverage) vs. natural infection (a measure of disease burden). Advances in antigen-level multiplex serology and proper antigen choice could be a solution to address this need.

### 3.3. Challenges in Using Serology-Based Techniques to Define Disease Burden

While serology is a powerful and promising tool for assessing antigen exposure, there are several factors that must be considered while interpreting serological data.

#### 3.3.1. Heterogeneity of Immune Responses

First, antibodies detected in an individual may have been induced by either infection (with or without symptoms), vaccination, maternal transfer (for young children) or a combination of these events. Disentangling the origin of detected antibodies can be challenging. A good understanding of the antigen-specificity of antibodies induced after infection vs. vaccination is crucial since prevalence of antibodies induced by infection indicates disease burden, while antibodies induced by vaccination can be a proxy for vaccine coverage. Some antibody-detection technologies are now able to reliably discriminate between antibody signatures induced by infection vs. vaccination, as for example routine Hepatitis B serology [42].

Secondly, not all pathogens induce seroconversion upon infection [21,23], and seroconversion rates can vary between individuals due to genetic and metabolic heterogeneity [43]. For example, acute infections such as measles, rubella and smallpox induce lifetime seropositivity that is a clear sign of past exposure. However, seroconversion is less consistent after human papillomavirus, rotavirus or typhoid infection, which do not always induce sustained and measurable antibody responses [21,23]. Moreover, antibody responses wane over time, with decay rates specific to the pathogen, vaccine and individual. In summary, while the presence of antibody is a sign of past antigen exposure or infection, the absence of detectable antibody cannot rule out previous exposure.

Finally, extrapolating antibody levels in the population to immunity levels or protection gaps is not always feasible. Indeed, protection from infection can only be inferred from serology for pathogens which have an established antibody concentration that confers to protection (a correlate of protection), such as for example hepatitis B, measles, rubella, smallpox and yellow fever [44]. On the other hand, presence of antibody against tuberculosis or malaria, for example, confirms previous infection but does not correlate with protection against subsequent infection [23].

#### 3.3.2. Measurements, Thresholds and Quantifying Immune Responses

Serology-based assays are inherently quantitative because the strength of the signal measured (readout) is proportional to the amount of antibody that is bound to a target. The first complication is that different assays measure different types of signal (e.g., optical density for enzyme-linked immunosorbent assay, color of a band for lateral flow rapid tests, luminescence for electro-chemiluminescence immuno-assay, see Section 4). For each assay, the intensity of the signal depends on avidity, valency, as well as the nature and concentration of both target and antibody. Therefore, connecting the numerical value of the readout, which are assay-specific and often given in “arbitrary units”, to an international standard representing an understood antibody quantity is not trivial and requires the use of established reference standards which are typically only available for vaccine-preventable diseases [20,22].

For public health programs, it is sometimes appropriate and sufficient to report qualitative results for serology-testing by categorizing individuals as either positive or negative for antibodies of interest (i.e., “seropositive” and “seronegative”). However, this classification requires an established cut-off value above which individuals are considered positive. This can be challenging and implies that assays have sufficient signal-to-noise ratio. The common methods to establish the seropositivity cut-off include the use of presumed unexposed populations, mixture models, receiver operating curves (ROC) and comparison to an international standard [22,43]. Setting a clear and widely adopted cut-off value is particularly important for comparability with other studies.

Finally, cross-reactivity between related or antigenically similar pathogens can cause false-positive signals and reduce specificity of certain assays. This is of particular concern in areas of co-endemicity of related viruses [26]. This phenomenon is well documented for flaviviruses [45,46,47,48], SARS-CoV-2 and seasonal common cold coronaviruses [22,49]. In addition, some pathogens induce polyclonal B-cell stimulation (e.g., Epstein-Barr virus, malaria) and induce false-positive antibody responses, particularly IgM, to non-related pathogens [46,50,51]. Careful selection of target antigens in serology-based assays may help to improve specificity.

#### 3.3.3. Data Analysis and Interpretation

Interpretation of serology data is complex because serology-based assays have varying degrees of sensitivity, specificity, background signal and cross-reactivity. In addition, immune responses vary between individuals and wane over time. Antibodies can be induced by infection, vaccination, maternal transfer or a combination of these. Therefore, complex mathematical models relying on numerous assumptions are often necessary to correlate serology data with disease burden [52,53,54], force of infection (i.e., the proportion of susceptible children seroconverting each year) [55,56,57,58] or proportion of previously infected in a population [59,60]. However, advances in statistical techniques and wider availability of statistical software have made these methods increasingly accessible [60]. For example, the R-package “sero-incidence” uses population level (cross-sectional) antibody data to generate an estimate of the frequency with which seroconversion occurs in the population [53].

## 4. Current Methods, New Technologies and Future Directions

**Figure 1:**
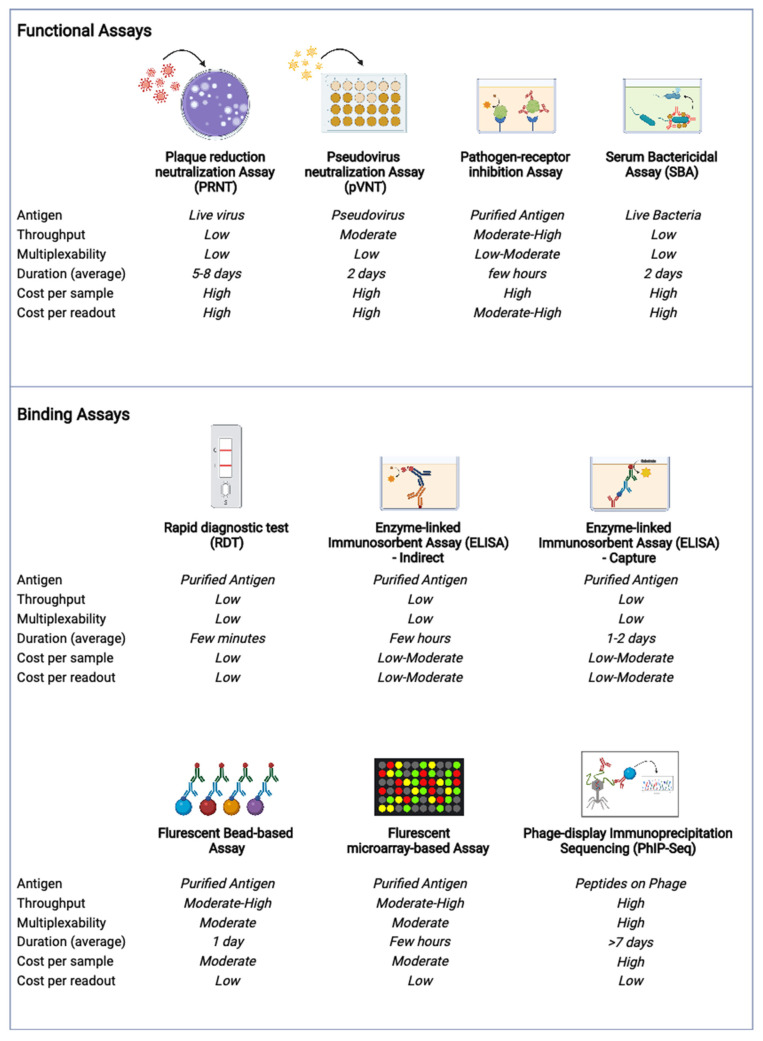
Summary of some of the current serological methods and their technical characteristics. Figure created using biorender.com.

Serological identification of pathogen exposure can be assessed using a variety of either binding or functional antibody assays (Figure 1). Assays measuring functional properties of antibodies (such as those measuring virus neutralization, inhibition of virus binding to host receptor, antibody dependent complement killing of bacteria [61]) are frequently more pathogen-specific. In some contexts, functional antibody assays can even differentiate between viral serotypes or variants, such as in dengue virus [62] or with SARS-CoV-2 [63], respectively. For viruses, functional properties such as antibody neutralization are frequently assessed through experimental techniques using live virus (such as plaque reduction neutralization assay (PRNT), focus reduction neutralization assay (FRNT), virus neutralization assay (VNT), fluorescent virus neutralization assay (FVNT)), inhibition of virus binding to host receptor (such as surrogate neutralization assay (sVNT)), or pseudovirus neutralization assay [64,65,66]. For bacteria, functional assays measuring antibody dependent killing of bacteria (such as bactericidal complement assay and bacterial phagocytosis assays) are more commonly used [67,68]. However, due to their time-consuming, expensive and low throughput nature, functional assays are not often used for surveillance purposes.

Experimental platforms that measure pathogen-specific antibodies through binding to pathogen-specific antigens are more widely utilized for infectious disease sero-surveillance and sero-epidemiology studies. Traditional tools such as enzyme-linked immunosorbent assays (ELISA), chemiluminescent immunoassay (CLIA) and lateral flow rapid diagnostic tests (RDT) are commercially available for various pathogens and frequently used in hospital settings for quick diagnosis of infectious diseases [22,26,69]. There also exist modified versions of ELISA assays using various non-enzyme-linked detections, such as fluorescent probes and chelators (e.g., time-resolved immunofluorescent assays) [70]. Three main forms of ELISA assays are frequently used to detect pathogen-specific antibodies, i.e., indirect ELISA, sandwich ELISA and capture ELISA [71]. Both indirect and sandwich ELISA formats are used to measure pathogen-specific IgA or IgG in convalescent disease (i.e., after infection and disease resolution), while capture ELISA is predominantly used to assess pathogen-specific IgM during acute infection and disease. Both ELISAs and RDTs are commonly conducted in LMICs as their implementation requires minimal equipment, resources and training.

The future of sero-diagnostics for surveillance and epidemiology are highly multiplexable technologies, i.e., experimental assays that can measure antibodies to numerous pathogens simultaneously. The most widely used multiplex serological platforms are the fluorescent bead-based assays and the microarray-based assays [72,73,74]. Both bead-based (such as Luminex immunoassays) and immune microarrays based serological assays can be conducted with small sample quantities and allow for multiplexing from tens to hundreds of pathogens. Due to high cost, bead and microarray-based multiplex serology assays are less accessible to low resource settings than ELISA techniques.

The newest cutting-edge multiplex serology assay with the potential to revolutionize sero-surveillance of infectious diseases is the phage-immunoprecipitation assay (PhIP-Seq) [41,75]. The PhIP-Seq technology allows serology to be “megaplexed”, i.e., the detection of up to a million different pathogen proteins. This assay has already been successfully run with various antibody-containing biological samples and used to study sero-prevalence of viruses and bacteria [76,77]. PhIP-Seq combines serology testing with next generation sequencing (NGS) for antibody detection. Unfortunately, molecular sequencing is currently too expensive to set up in low resource settings. Furthermore, PhIP-Seq requires trained personnel for both the experimental protocol and the subsequent bioinformatic analysis. However, as NGS and PhIP-Seq becomes more widespread in LMICs hopefully these financial and skill-based barriers can be overcome. The integration of multiplex serological techniques, such as PhIP-Seq, into surveillance programs (including the different sectors of One Health) could enable simultaneous tracking of numerous pathogens and possibly even the detection of novel pathogens before a disease outbreak.

## 5. Real-Life Examples and Public Health Use

### 5.1. Serology to Guide Child Health Policies and Vaccine Roll-Out

Recently, a study in the Netherlands used respiratory syncytial virus (RSV) serology (RSV-specific IgG and IgA directed against five different antigens) in two cross-sectional surveys to classify children under five as seronegative or seropositive [78]. Subsequently, generalized additive models were used to assess the probability of past RSV infection as a function of age, date of birth during the year and other risk factors of interest. The study showed that by two years of age, the majority of children had been infected at least once.

Because serology is particularly appropriate for multiplexing, this approach could be expanded to simultaneously detect antibodies to multiple pathogens of interest in children of different ages. This age-stratified sero-prevalence data could inform health authorities about the relative burden of various infectious diseases in a population and estimate the risk of exposure as a function of age. This type of information could guide prevention measures for children in their first year of life including helping public health authorities optimize vaccine scheduling and immunization strategies.

### 5.2. Serology to Complement Case-Based Clinical Reporting

Cholera surveillance is another example where serology has been used [52]. Traditionally, information on cholera incidence is based on reporting of acute watery diarrhea but often without laboratory confirmation. Azman et al. developed a model based on either six or two antibodies against various vibrio cholera targets to identify individuals with recent cholera infection. The models were built using machine learning (random forest) on a cohort of known cases and household controls from Dhaka. Both models correctly identified individuals who had suffered cholera in the past year. The two-antibody model was validated on a cohort of North American volunteers that had been experimentally challenged with cholera. The authors mentioned that while clinical surveillance would continue to play a crucial role to understand clinical, microbiological and resistance trends, serology in cholera hotspots during or after outbreaks could help better define the extent of temporal and geographical transmission, regardless of care-seeking behavior or reporting system failures.

This example illustrates how serology could complement classical case-based surveillance, in low-resource settings where (i) etiology is not routinely confirmed by a laboratory test, (ii) health-seeking behavior or access to health care may prevent the health-system to capture cases or (iii) reporting and data sharing frameworks are not well established. As previously mentioned, multiplexing could be an efficient way of generating valuable information on the burden of multiple pathogens of interests simultaneously in regions where passive surveillance is either not established or not feasible due to limited resources. Another advantage is that a cross-sectional survey to understand sero-prevalence can be performed as a “one-off” activity, unlike case-based surveillance which needs to be maintained over a period of time to generate sufficient information.

### 5.3. Serology to Assess the Burden of Infection beyond Symptomatic Cases

Direct detection of dengue cases can be complicated by the fact that (i) dengue infections are often asymptomatic, (ii) when symptomatic, clinical signs and symptoms can be very similar to other arboviruses such as Zika or Chikungunya and (iii) viremia can be short lived or decreasing when symptoms appear, opening only a short window for molecular confirmation [56,79,80]. For all these reasons, dengue surveillance is another field in which serology has been informative.

A recent study used seroprevalence data (IgG ELISA and neutralization titers) from healthy children in 46 distinct sites in 13 countries to describe dengue endemicity across a wide spectrum of regions [56]. If transmission intensity is assumed to be stable over time, age-stratified sero-prevalence can be used as an indicator of endemicity by calculating the force of infection (FOI), i.e., the rate at which susceptible individuals acquire infection.

Another study in the Solomon Islands used a cross-sectional survey to detect IgG for four viruses with overlapping symptoms (Dengue, Zika, Chikungunya and Ross River Virus) and to establish their relative burden [80]. The results showed that Dengue and Ross River Virus had the highest burden on the islands and highlighted the presence of Ross River Virus, even though cases had not been reported prior to the study.

Both studies exemplify how serology can be instrumental to estimating disease burden for pathogens that frequently cause asymptomatic infections. This is important because asymptomatic cases often contribute to transmission as seen for dengue, influenza, measles and COVID-19 [81,82]. Therefore, asymptomatic cases should be taken into consideration when prevention measures are planned. Similarly, if serology is specific enough to differentiate between pathogens of interest, it can help estimate the relative burden of diseases that have overlapping symptoms, and therefore cannot be distinguished based on clinical presentation alone. Finally, serology can highlight the circulation of certain pathogens even in the absence of reported cases.

### 5.4. Serology to Measure Impact of Public Health Interventions

Due to the long-lasting nature of most antibody responses, and the fact that the presence of antibody is not always easily linked to the timing of infection, it is arguably more difficult to use serology to measure impact of public health interventions. Despite these limitations, serology has been used to assess the impact of long-lasting insecticidal bed nets distribution on malaria and filariasis occurrence in Mozambique [83]. In this study, dried-blood spots taken before bed net distribution and one year later were probed by multiplex serology for the presence of IgG antibodies to six malaria and three lymphatic filariasis antigens. Over the study period, seroconversion rates for malaria and lymphatic filariasis were compared in households with and without bed nets. This study relied on the fact that malaria seropositivity rates correlate with intensity of malaria transmission, at least in low endemicity regions [84]. Overall, seropositivity to malaria and lymphatic filariasis decreased for several antigens over the study period, but bed net use was associated with reduced seropositivity for two of the malaria antigens only. These examples suggest that serology could be used to monitor the impact of some interventions, but perhaps best in combination with other more direct indicators of disease burden.

### 5.5. Serology during the COVID-19 Pandemic

Serological methods have been extensively used during the COVID-19 pandemic to answer a wide range of questions. Before the pandemic, serology was used to track potential spill-over from viruses of bat origin into humans in Southern China [85]. In this study, serum samples from individuals living in rural areas where coronaviruses had been identified in bats were analyzed by ELISA for the presence of IgG for four bat coronaviruses. Individuals with high exposure to bats and other wildlife were selected for the study. Bat coronavirus antibodies were detected in a low number of participants. This observation confirmed previous exposure and provided evidence for bat coronavirus spill-over to humans in rural Southern China, although at a low frequency.

This type of early-warning serological surveillance could be used to detect potential emergence of zoonotic disease, characterize viral sequences and identify high-risk populations prior to large-scale outbreaks [85,86]. In the future, such investigations could function as early warnings and hopefully reduce the risk of (and help manage) outbreaks although careful consideration and selection of the most relevant pathogens as well as reservoirs would be crucial to guide such screening efforts. Potentially, this approach could be used to probe samples from blood banks periodically to detect spill-over events from animal reservoirs or capture the expansion of known pathogens to new populations or regions.

**Figure 2:**
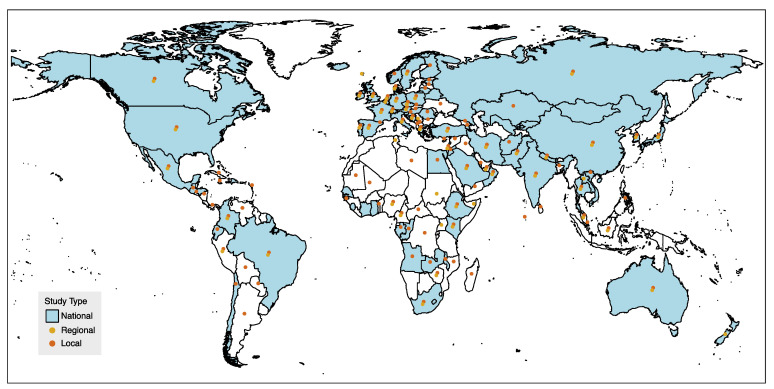
Countries that performed national, regional and/or local sero-surveys for COVID-19. The placement of the dot does not represent the location of the study, but simply indicates the country. Based on SeroTracker data.

During the COVID-19 pandemic, serologic studies have proven crucial to understand the amount of past infection, which is necessary to predict future transmission patterns [59,60], monitor the pandemic and guide public health responses [87,88,89]. Early on, the WHO proposed a standard protocol for population-based, age-stratified sero-epidemiological studies for assessing SARS-CoV-2 infection and estimating key parameters such as seroprevalence of antibodies to COVID-19, cumulative incidence of infection, attack rates, proportion of asymptomatic infection and case fatality ratio [90]. Rapidly, countries expressed their interest, and sero-surveys were implemented across the world. The SeroTracker initiative was established to synthetize the results of sero-epidemiological studies worldwide in real-time and visualize sero-prevalence estimates in an online dashboard [87]. A map of the world depicting which countries have performed COVID-19 sero-surveys to date is presented in Figure 2.

## 6. Conclusions

Both case-based detection and serology can provide crucial information about infectious disease burden and local epidemiology to guide public health decisions. Both approaches have strengths, limitations and specificities that determine how they can be implemented, and in which settings (Table 1). Overall, and when possible, combining pathogen detection and serology methods may generate the most exhaustive description of the disease landscape [24] to guide public health decisions.

## Figures and Tables

**Table 1:** Comparison between case-based and serology-based surveillance.

	Case-Based	Serology-Based
Cases detected	Current/ongoing Symptomatic (mainly)	Past Symptomatic/asymptomatic
Health system presentation	Necessary ^(^*^)^	Not necessary
Time window for detection	Short (days)	Medium-Long (month-years)
Possible on stored samples	Rarely	Yes
Possibility of multiplexing	Yes	Yes
Challenges to implementation in low resource settings	Healthcare access barriers Limitations in use of confirmatory diagnostics	Financial and infrastructure constraints depending on test chosen

^(^*^)^ except in systematic detection in contact tracing activities or active surveillance.

## Data Availability

Publicly available data from Serotracker can be found under: https://serotracker.com/en/Data.
